# Supplementary material for: Extracting information from RNA SHAPE data: Kalman filtering approach
Source: PLoS One. 2018 Nov 21;13(11):e0207029. doi: 10.1371/journal.pone.0207029 (PMC6248965; doi:10.1371/journal.pone.0207029)
Supplement: S1 Text — (PDF) [file pone.0207029.s013.pdf]

S1 Text for "Extracting information from RNA SHAPE data: Kalman filtering approach"

Normalization of simulated SHAPE replicates

Currently, there is no standardized practice for normalizing a given SHAPE profile [1] and, beyond this, the heuristic methods typically applied in practice have received criticism [2]. Replicates that were simulated under the assumptions of the log-normal noise model as described in Methods were assumed to be post-normalization. However, we were interested in investigating whether performing an additional normalization step would have any effect on the relative results of the log-averaging and Kalman filtering processing methods. We refer to the additional normalization step as a *re-normalization* step. Additionally, given the discord on normalization techniques, we were interested in studying the effects of outlier exclusion on the normalization process and downstream analysis. We provide a study on these two points below.

Before proceeding, we would like to highlight one of the difficulties of working in the log domain. Although replicates were both simulated and processed in the log domain, applying normalization in the log domain can be problematic. Intuitively, the log transformation changes the spread of reactivities, and in particular compresses higher valued reactivities. This reduces the percentage of outliers. Performing a normalization in the log domain would effectively result in data domain reactivities lying far outside the typical expected range of values (between 0 and 2). In the log domain, after the removal of outliers, the normalization factor may be less than 1 and, in fact, can be negative. Normalizing the log measurements by a factor less than 1 will result in an increase in the log measurement values. A 2-fold increase in a log measurement will ultimately result in 10-fold increase in the data domain. Thus, normalization of replicates in the log domain is impractical.

On the other hand, reverting the simulated replicates to the data domain, performing normalization, and re-applying a log transformation may result in a violation of the assumption of log-normality in the measurements. To illustrate this, suppose N replicates in the log domain l^1, l^2, $\dots l^N$ were simulated under the log-normal noise model, as described in Methods . In the data domain, the replicates are denoted r^1, r^2, $\dots r^N$. In practice, normalization is applied to each replicate separately in the data domain. Thus, the ith replicate is scaled by normalization factor c_i. Recall a nucleotide m then has measurements r^1_m, r^2_m, $\dots r^N_m$ and log measurements l^1_m, l^2_m, $\dots l^N_m$, where $l^i_m = \log(r^i_m)$. The ith normalized data domain measurement is $c_i r^i_m$. In the log domain, the ith normalized log measurement, l'^i_m, is

$$\begin{aligned} l'^{i}_{m} &= \log(c_i r^i_m) \\ &= \log(c_i) + \log(r^i_m) \\ &= \log(c_i) + l^i_m \end{aligned}$$

Thus, by applying Eq 3 of the main text, the l'^{i}_{m} normalized log measurement is related to the ground truth log measurement l_m by the relationship $l'^{i}_{m} = \log(c_i) + l_m + w^i_m$. The constant, $\log(c_i)$, is different for each replicate. This violates the assumption of additive zero-mean Gaussian noise in log-normal noise model and thus renders both log-averaging and the Kalman filtering inapplicable to the problem. Nevertheless, we studied the relative results of the two filtering methods after re-normalization below.

Effects of replicate normalization on simulated SHAPE reactivity ranges

We start this section by noting that if the reactivity distribution in a replicate is significantly different from the expected prior, then using such a prior distribution will introduce a bias in Kalman filtering results (see Results on refining the Kalman filter prior). The purpose of normalization is to ensure the range of reactivities is within the typical range (0 to about 2 for SHAPE data). If the data range is significantly different from this, we expect to see a loss of accuracy in Kalman filtering results. To study the effects of re-normalization on simulated replicates reactivity ranges, we first simulated 3 replicates for all 22 RNAs in our database (see Table 1 in the main text). For each RNA, we then carried out the following two normalization processes in the data domain:

1. **Normalization 1:** For each replicate, the top 10% of the most highly reactive nucleotides (including outliers) were averaged to compute the normalization factor. The entire replicate was normalized by this factor.

2. **Normalization 2:** For each replicate, the interquartile range (IQR) was first calculated. Outliers were defined as being greater than 1.5xIQR above the upper quartile [3]. As in [4], the number of outliers was capped at 10% for RNAs at least 100 nucleotides long and 5% for shorter RNAs. For the remaining nucleotides, the top 10% of the most highly reactive nucleotides were averaged to compute the normalization factor. The entire replicate, including outliers, was normalized by this factor.

The two normalization methods described above differ in that the first method does not exclude outliers from the calculation of the normalization factor. As discussed in Background section, outliers are most often excluded, as in Normalization 2. We

performed Normalization 1 in order to study the effects of outlier exclusion. We thus considered three sets of replicates:

1. **SET0:** The original simulated replicates.
2. **SET1:** The replicates modified under Normalization 1.
3. **SET2:** The replicates modified under Normalization 2.

We repeated this for replicates simulated at low, medium, and high noise levels. Box plots for the resulting reactivities of all RNA replicates combined are shown in S3 Fig. The range of values for the replicates in SET0 aligns well with those in SET2. Replicates in SET1 exhibited a range of reactivities smaller than what is typical in a SHAPE experiment. This highlights the importance of excluding outliers when calculating the normalization factor. The similarity in ranges for SET0 and SET2 agrees with our assumption that no additional normalization is required following replicate simulation.

Effects of normalization on processed SHAPE profiles

To illustrate how replicate re-normalization effects the profiles resulting from the different processing methods explored, we first performed a simple case study. For a particular RNA, we simulated 3 medium-noise replicates, as described in Methods. We then performed Normalization 1 and 2, as described above. We calculated the average, log-average, and Kalman filter profiles on each of the resulting sets of replicates (SET0, SET1, and SET2, described above). The results shown in S4 Fig - S6 Fig are for the following RNAs: TPP riboswitch, *E. coli*, Group I intron, *Azoarcus sp.*, and Hepatitis C virus IRES domain (original SHAPE profiles for all 3 RNAs are from [4]). The profiles obtained by processing SET1 exhibited the most disparity compared to the ground truth SHAPE profile. RMS values calculated in the log domain also indicate that, for all processing methods, profiles calculated using the original simulated replicates in SET0 agreed most with the ground truth.

Normalization does not affect comparisons between log-averaging and Kalman filtering

After applying a particular re-normalization scheme, we were interested in the comparison between the log-averaging and Kalman filtering denoising methods. We performed the following simulations:

1. For all 22 RNAs in our database (see Table 1), we first simulated 3 replicates, as described in Methods.
2. We reverted the log replicates to the data domain by applying an exponential transformation.

3. We modified the replicates under both Normalization 1 and 2 in the data domain.
4. For both sets of replicates, we performed log-averaging and Kalman filtering.
5. We simulated 10 replicates for each RNA and repeated steps 1-4.
6. We calculated RMS errors and recreated the heat maps of Fig 4 for both normalization techniques.

The results for replicates modified under Normalization 1 and 2 are shown in S7 Fig and S8 Fig respectively. As in the results of Fig 4, we observe that even if replicates are re-normalized, in the higher noise regimes, Kalman filtering recovered better the ground truth reactivity than did log-averaging. Similarly, after increasing the number of replicates to 10, the advantage Kalman filtering provides over log-averaging is not as drastic.

As a final test, we recreated the results of Fig 5 as follows. We first simulated from 2 to 10 replicates and modified the replicates under both Normalization 1 and 2. We then performed log-averaging and Kalman filtering on the updated replicates. We performed this simulation for replicates generated at low, medium, and high noise levels for all RNAs in our database. The RMS errors for log-averaging and Kalman filtering methods are shown in S9 Fig plotted against the number of replicates for replicates modified under Normalization 1. Similarly, the results in S10 Fig were generated for replicates modified under Normalization 2. The results produced using replicates modified under Normalization 2 (S10 Fig) mirror those produced using the original replicates (Fig 5). The results for replicates modified under Normalization 1 are less intuitive: although the Kalman filtering approach better recovers the ground truth compared to log-averaging, its performance suffers as the number of replicates increases. We reiterate that 1. modifying the replicates under either Normalization 1 or 2 violates the assumptions made by the Kalman filter (see Methods and the above discussion on how re-normalization violates the assumption of the log-normal noise model) and 2. replicates modified under Normalization 1 resulted in reactivity ranges far below the expected range of 0 to 2, as illustrated in S3 Fig. Thus, for replicates modified under Normalization 1, not only is the Kalman assumption violated, but the prior used by the filter is also inaccurate. Additionally, both log-averaging and Kalman filtering produces significantly more errors compared to results calculated using the original replicates (Fig 5) and those modified under Normalization 2 (S10 Fig). Despite the heuristic nature of normalization methods, the exclusion of outliers is critical. However, note that the prior distribution used in our analysis was modeled on a database made up of profiles normalized excluding outliers, as in Normalization 2 (see Table 1 and a description of database in Methods). It is possible to alternatively model a prior based on data that has been normalized by any technique of choice. This would improve the Kalman filtering results for replicates normalized by the same technique.

References

1. Choudhary K, Shih NP, Deng F, Ledda M, Li B, Aviran S. Metrics for rapid quality control in RNA structure probing experiments. Bioinformatics. 2016;32(23):3575–3583. doi:10.1093/bioinformatics/btw501.

2. Eddy SR. Computational Analysis of Conserved RNA Secondary Structure in Transcriptomes and Genomes. Annual Review of Biophysics. 2014;43:433-456. doi:10.1146/annurev-biophys-051013-022950.

3. Sloma MF, Mathews DH. Improving RNA Secondary Structure Prediction with Structure Mapping Data. In: Chen SJ, Burke-Aguero DH, editors. Methods in Enzymology. vol. 553. Waltham: Elsevier; 2015. p. 91–114. doi:https://doi.org/10.1016/bs.mie.2014.10.053

4. Hajdin CE, Bellaousov S, Huggins W, Leonard CW, Mathews DH, Weeks KM. Accurate SHAPE-directed RNA secondary structure modeling, including pseudoknots. Proceedings of the National Academy of Sciences. 2013;110(14):5498–5503. doi:10.1073/pnas.1219988110.
